# Nanotechnology Scaffolds for Alveolar Bone Regeneration

**DOI:** 10.3390/ma13010201

**Published:** 2020-01-03

**Authors:** Goker Funda, Silvio Taschieri, Giannì Aldo Bruno, Emma Grecchi, Savadori Paolo, Donati Girolamo, Massimo Del Fabbro

**Affiliations:** 1 Department of Biomedical, Surgical and Dental Sciences, University of Milano, 20122 Milan, Italy; silvio.taschieri@unimi.it (S.T.); aldo.gianni@unimi.it (G.A.B.); massimo.delfabbro@unimi.it (M.D.F.); 2 IRCCS Orthopedic Institute Galeazzi, Via Riccardo Galeazzi, 4, 20161 Milano MI, Italy; paolo_savadori@yahoo.it; 3 Dental and Maxillo-Facial Surgery Unit, IRCCS Ca Granda Ospedale Maggiore Policlinico di Milano, Via Francesco Sforza 35, 20122 Milan, Italy; emma.grecchi@gmail.com; 4 ASST Fatebenefratelli Sacco Hospital, Dentistry Department, Via Giovanni Battista Grassi, 74, 20157 Milan, Italy; girolamo.donati@asst-fbf-sacco.it

**Keywords:** scaffolds, nanomaterials, tissue engineering, regenerative medicine, alveolar bone regeneration

## Abstract

In oral biology, tissue engineering aims at regenerating functional tissues through a series of key events that occur during alveolar/periodontal tissue formation and growth, by means of scaffolds that deliver signaling molecules and cells. Due to their excellent physicochemical properties and biomimetic features, nanomaterials are attractive alternatives offering many advantages for stimulating cell growth and promoting tissue regeneration through tissue engineering. The main aim of this article was to review the currently available literature to provide an overview of the different nano-scale scaffolds as key factors of tissue engineering for alveolar bone regeneration procedures. In this narrative review, PubMed, Medline, Scopus and Cochrane electronic databases were searched using key words like “tissue engineering”, “regenerative medicine”, “alveolar bone defects”, “alveolar bone regeneration”, “nanomaterials”, “scaffolds”, “nanospheres” and “nanofibrous scaffolds”. No limitation regarding language, publication date and study design was set. Hand-searching of the reference list of identified articles was also undertaken. The aim of this article was to give a brief introduction to review the role of different nanoscaffolds for bone regeneration and the main focus was set to underline their role for alveolar bone regeneration procedures.

## 1. Introduction

The reconstruction and augmentation of the alveolar bone is a complex and challenging field for the maxillofacial and periodontal surgeon. The crucial aim of the therapies in this field is mainly increasing the bone mass in patients who have lost this tissue as a result of a consequence of several conditions such as periodontal disease, aging, osteoporosis, trauma, neoplastic pathology and reconstructive surgery or as a result of congenital defects [1].

At present auto-transplantation from a patient’s extra-oral or intra-oral donor site is accepted as the gold standard and is the most frequently used method [2]. The critical limitations of this conventional approach are donor site morbidity, inadequate blood supply of graft tissue, associated pain and limited supply. For these reasons, autologous grafting is usually reserved for a restricted number of cases [2,3].

Alternative sources for bone grafts include allografts (grafts originating from another individual of the same species) and xenografts (grafts originating from different species such as bovine or porcine). However, these substitutes also have some certain disadvantages such as the possibility of immune rejection and pathogen transmission from donor to host [2,3].

Another strategy for bone grafts is the use of synthetic alloplasts made from polymers, ceramics or metals. Alloplasts represent some disadvantages including non-optimal integration with the native tissue at the site of the defect and the potential failure due to fatigue or infection caused during implantation. In addition to these, their applications are limited at locations of high stress or mechanical load [2,3].

Owing to the drawbacks and limitations of bone grafts, over the last few decades, several novel approaches involving tissue engineering and regenerative medicine (TE/RM) have emerged as alternatives to conventional treatments. The fundamental concept underlying TE/RM was to use scaffolds alone or in combination with growth factor, cell and/or gene delivery to form a so-called “tissue engineering construct,” that stimulates tissue repair and/or regeneration [2,3,4,5].

In brief, the four crucial factors that must be considered with TE/RM are:

- Cells, which represent the fundamental structural unit of any tissue,
- A matrix such as “scaffolds”, as framework material supporting the growth of cells to form a fully organized tissue,
- Biological factors like growth factors (GF) and bone morphogenetic proteins (BMPs) to guide cellular activity and tissue formation,
- Vascularization to provide oxygen and nutrients for the cell metabolism, and to remove catabolic waste products [6,7].

Current limitations in bone TE/RM strategies are the impaired cellular differentiation, inadequate synthesis of extrinsic factors essential for an effective osteogenesis and, most importantly, insufficient mechanical strength and physicochemical properties of the conventional scaffolds [7]. Conventional scaffolds combined with growth factors and cells do not always achieve successful bone regeneration in clinical settings, generally due to the limited capability of controlling framework degradation as well as delivery of biological factors and drugs [6,8,9].

The focus of this review was to give a brief introduction to the nanoscaffolds in TE/RM and to underline the role of different scaffolds in successful tissue formation for bone regeneration. The main aim of this literature-based article is to provide an overview of the different nanoscale scaffolds as key factors of the tissue-engineering paradigm, used for alveolar bone regeneration.

In order to find articles pertinent to this narrative review, PubMed/Medline, Scopus and Cochrane electronic databases were searched using key terms such as “tissue engineering”, “regenerative medicine”, “alveolar bone defects”, “alveolar bone regeneration”, “nanomaterials”, “scaffolds”, “nanospheres” and “nanofibrous scaffolds”. No limitation regarding study design, publication date and language was set. A hand search through the references of the identified articles and previous reviews was also undertaken. The last electronic search was performed on 3 December 2019.

### 1.1. Importance of Scaffolds in TE/RM

Scaffold material plays a key role in tissue regeneration, as it provides a micro-environment suitable for cell adhesion, proliferation and differentiation. In general, an ideal scaffold material should be biocompatible, have a controllable degradation and appropriate physico-chemical features in order to simulate the extracellular matrix (ECM) structure of the original tissues [7]. It should be able to balance various combinations of materials with specific functions by means of engineered surface, cell encapsulation and controlled release of chemicals [6,9]. Additionally, it should be able to endorse and control peculiar events occurring at the cellular and tissue level [10].

In scaffold based tissue engineering, the scaffold is expected to perform several functions. It should provide adequate mechanical strength and stiffness to replace the mechanical properties of the missing or the injured tissue [6,11]. Most importantly, a successful scaffold should stimulate not only the early ingrowth of new tissue, but also the progressive maturation and remodeling by providing adequate support and morphology [12]. Its design should also take into account degradation kinetics and physico-chemical features [6,11].

### 1.2. Nanotechnology

One thousand nm is equal to 1 micrometer (µm) and nanoparticles are smaller than 100 nanometers (nm). Nanomaterials compared with bulk materials possess features like quantum size, macroscopic quantum tunneling, as well as small size effects, resulting in altered physiochemical properties [7].

Current areas of research in nanotechnology for tissue regeneration are as follows:

- Nanoparticle-based techniques for delivering bioactive molecules,
- Nanoparticle-mediated cells tagging and targeting,
- Nanoparticle-based scaffold manufacturing [7].

Additionally, the half-life and distribution of nanoparticles can be affected by their size. Their surface properties can determine their stability and their localization in the tissues [7]. A decreased size in nanomaterial particles is beneficial in terms of stiffness, effective surface area and area-to-volume ratio [7]. The charge of the nanoparticles is also another important factor affecting the internalization of nanosized particles into different target cells [7].

In oral biology, nanotechnology applications are mainly focused on augmentation procedures for bone tissue regeneration and enhancement of osseointegration of oral implants [7]. Natural bone itself is constituted by a highly organized extracellular matrix (ECM) in a nanometric scale, and the application of nanoscaffolds can represent an intrinsic advantage for tissue engineering regeneration procedures of the musculoskeletal apparatus [8,13]. Nanomaterials can overcome the main problems encountered with the current scaffolds used for bone regeneration such as: Inadequate mechanical strength, instability of growth factors released and impaired cellular differentiation [7,10].

### 1.3. Rationale of Nanotechnology Scaffolds in Tissue Engineering

Advanced materials play a crucial role in promoting the bench-to-bedside translation of tissue engineering technologies [14,15]. Direct application of therapeutic substances may be affected by some limitations of the conventional scaffold materials including non-specific targeting, insufficient physiological stability and low cell membrane permeability. Usually, supra-physiological doses are needed to compensate for the poor pharmacokinetics of such agents, which in return increase the potential risk of adverse effects [7]. Currently, nanotechnology has allowed the production of structures having the same size as the naturally occurring tissues and has opened a new era for TE/RM [8,16].

Nanoscaffolds can be produced so as to be extremely similar to tissue-specific ECM. The reduced size of the nanoparticles permits a fast response to external stimuli coming from the environment, like ultrasounds, magnetic fields, pH and iX-ray exposure.

Nanoscaffold materials may be used to deliver drugs, genetic material or biological factors in a controlled way, both systemically and locally [8]. Nanoscaffolds can stabilize the bioactive agents by means of encapsulation or surface attachment, promoting molecule internalization, targeting their delivery from cells and allowing to control biological factor release at the intended target [7]. Controlled and sustained delivery by nanoparticles mainly depends on their reduced size and related high specific surface area [17]. Thus they may represent stimulus-sensitive delivery vehicles for chemically or biologically active substances, which will provide a triggered delivery as a response to an external stimulus [7,16,17].

Nanoscaffolds have a considerable drug loading capability, high mobility of drug loaded particles, and efficient in vivo reactivity toward nearby tissues [17]. They can be used for labeling cells, in order to enable continuous cell tracking and monitoring [8,16]. Additionally, nanoparticles can provide enhanced osseointegration, osteoconduction and osteoinduction [16].

### 1.4. Nanofibrous Scaffold Systems

Fibrous nanoscaffolds are extremely-thin uninterrupted fibers with a short diffusional path, a considerable surface area to unit mass ratio and high porosity. The porous structure of the nanofibrous drug delivery systems is highly interconnected and represents an adequate substrate for cell adhesion and transport of nutrients. One of the limitations of these systems is the initial burst release phenomenon, which depends on the large surface area and the short diffusional path.

Fibrous nanoscaffolds, besides their role as drug carriers, provide mechanical strength. There had been worries that drug incorporation into the nanofibers might impair the mechanical features of the same nanofibers [8]. As an example for a solution to this problem, Ionescu et al. developed a microsphere-laden fibrous nanoscaffold structure in which the microspheres were to deliver drugs, while the nanofibers only worked as an engineered scaffold [18].

### 1.5. Nanosphere Scaffold Systems

Nanospheres can deliver drugs, growth factors or genetic material [7]. The advantages of nanospheres over conventional monolithic bulk scaffolds can be listed as follows:

- Mechanically weak scaffolds in load-bearing applications may be reinforced by the adjunct of nanospheres as cross-linking agents,
- The porosity of the materials constituting traditional scaffolds may be significantly enhanced, by adding spheres like porogen. Increased porosity means allowing interior tissue infiltration into the scaffold,
- Nanospheres can stimulate the creation of apatite crystals and the following mineralization of hydrogels through the release of the corresponding minerals, so that self-hardening biomaterials adapted to the regeneration of bone tissue can be produced.
- Injectable and/or moldable materials can be developed thanks to the spherical nature of some nanomaterials, so that their application is possible by means of minimally invasive surgery [17].

### 1.6. Classification of Nanoparticle (NP) Materials

**Figure 1:**
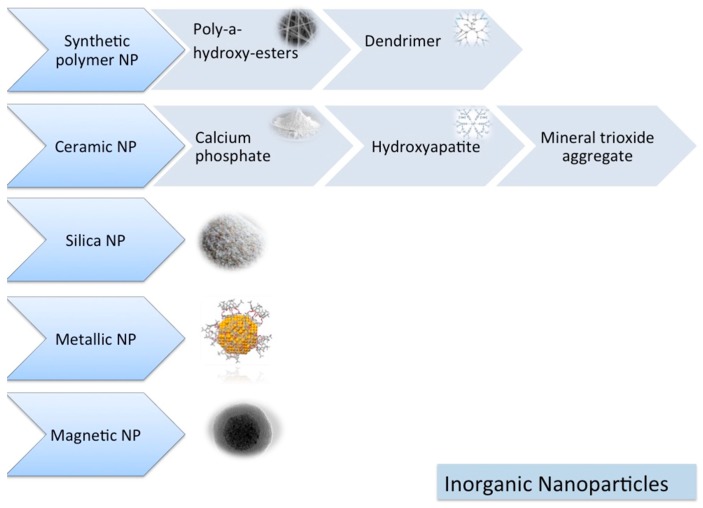
Inorganic nanomaterials.

**Figure 2:**
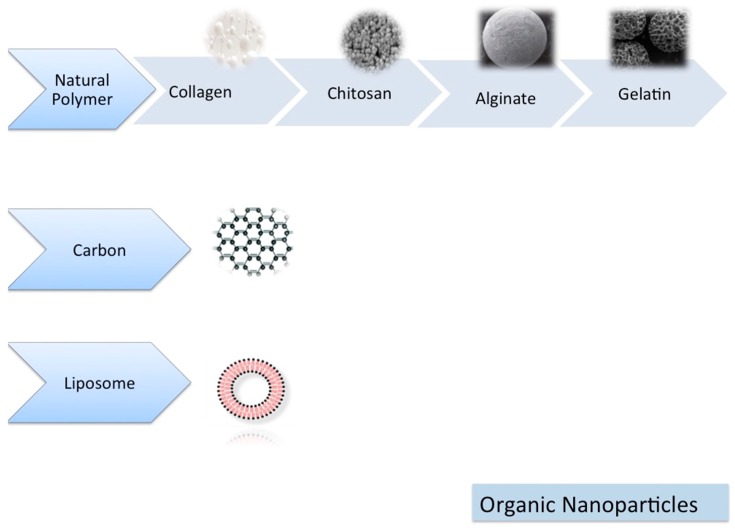
Organic nanomaterials.

Nanoparticles can be classified as inorganic (Figure 1) and organic nanoparticles (Figure 2).

#### 1.6.1. Inorganic NPs

##### Synthetic Polymers

Synthetic polymers have some advantages, such as sufficient supply, easy fabrication, easy adaptation, high safety profile and reasonable costs. Additionally, they have adjustable physiochemical and morphological features, which are valuable for wide-scale production and application [19,20].

The most common synthetic polymers for biomedical applications are poly-α-hydroxyesters (poly-glycolic acid (PGA), poly-lactic acid (PLA), poly-lactic-glycolic acid (PLGA) and poly-caprolactone (PCL)) [20]. However, there are also disadvantages of poly-α-hydroxyesters, which can be listed as follows: (i) Hydrophobicity, which causes failure of loading hydrophilic drugs or molecules and poor cell adhesion, (ii) degradation by autocatalysis, which causes unpredictable degradation behavior, (iii) an acidic degradation product, which leads to denaturation of bioactive proteins and inflammatory tissue response, and (iv) low capacity of loading therapeutic agents, which limits their penetration into the polymer network [20,21].

Various combinations of PLGA/PLLA/PEG/PCL were tested by several researchers with promising results for alveolar bone regeneration applications [1,22,23,24].

• Dendrimers

Dendrimers are synthetic polymers with branching treelike structures. They are biocompatible and biodegradable with uniform morphology. They have a high number of surface functional groups, which makes them suitable for several applications [16,25]. Dendrimers can cause an improvement on drug solubility [16,26] and are mostly used for targeted drug delivery reasons [16,27]. However, they have a few disadvantages, like cytotoxicity and possible poor drug retention inside the branches of the dendrimer [16].

##### Ceramic NPs

Ceramic materials are “synthethic crystalline, solid, inorganic non-metallic materials” [28]. Bioceramics include bioactive glass, bioactive glass–ceramic, calcium phosphate groups and alumina [16,29,30].

Bioceramics possess certain advantages like biocompatibility, nontoxicity and dimensional stability [29]. They are added to the scaffolds to improve their structural properties and delivery performance. Many ceramic materials such as calcium phosphate groups and mineral trioxide aggregate materials have been used and are currently in use for bone regeneration procedures [16].

Bioactive glass nanoscaffold was investigated and was reported to be beneficial for formation of new alveolar bone tissue [31].

• Calcium Phosphate (CP) Groups

Normal bone tissue is composed of 30% *w*/*v* organic collagen fibers and 70% inorganic matter, mainly CP crystals. The latter represents a model for mimicking natural bone material at the macro- and nanoscale level [7]. For this reason, CP nanoparticles (nano-tricalcium phosphate (nTCP) and nano-hydroxyapatite (nHA)) have been the most commonly applied materials for bone tissue regeneration [32].

CP has many favorable properties such as similarity to the inorganic portion of natural bone tissue, biocompatibility, biosafety, osteoconductivity, low cost and ease of manufacturing. The drawbacks are poor regulation of drug delivery and degradation rate [20,33]. However, when CP is used as a single component, it might represent some limitations due to its poor mechanical features and very low toughness. In order to overcome such drawbacks, researchers recently introduced composite scaffolds composed of nTCP/nHA plus further biological materials or synthetic polymeric materials [33].

Several applications of nanoHA [34,35,36,37,38,39] and nanoTCP [40,41] combinations were investigated in the literature and were reported as successful for alveolar bone tissue regeneration.

• Mineral Trioxide Aggregate (MTA)

MTA is prepared by bismuth oxide reaction, and is composed of tricalcium aluminate, tricalcium silicate, dicalcium silicate and calcium sulfate dehydrate [42,43]. During hydration, MTA releases Ca2+ and OH ions, which increases pH in order to neutralize the acidic metabolites produced by macrophages and osteoclasts. Nanoscale particle sized tricalcium aluminate may increase bone regeneration, inflammatory response and foreign body reactions [43].

##### Silica NPs

Silica, either itself or as a coating of other compounds, has been used for applications in the biomedical field, like imaging and drug delivery [16]. According to their application purposes, silica nanoparticles can be synthesized as bulk particles, core/shell silica NPs and mesoporous silica nanoparticles (MSNPs) [16]. In particular, MSNPs have attracted the attention of researchers for applications of controlled release. MSNPs can be synthesized in different ways to obtain particles with various dimensions and physical properties. MSNPs have unique pore structure, high surface area and large pore volume. Additionally, because of their honeycomb-like porous structure, they are able to encapsulate and absorb a number of biomolecules [16,44,45].

The typical advantages of silica nanoparticles are their uniform morphology, biocompatibility and chemical stability. Their disadvantage is their variable toxicity [16].

##### Metallic NPs

Metallic NPs are biocompatible materials with reduced cytotoxicity. Their functionalization is easy; however, they need long term cytotoxicity testing and their coating is advised [16]. In biomedical applications, gold NPs are considered as the safest and the most commonly utilized among other metallic NPs. Gold NPs can be synthesized as spheres, rods or cages and they can be applied in areas such as drug delivery, biosensing, bioimaging and photothermal therapy [16,46,47].

Combinations of gold and silver nanoparticles with chitosan for alveolar bone regeneration were reported for gold [48] and silver [22] with successful outcomes for enhanced mineralization and implant osseointegration.

##### Magnetic NPs

In biomedical applications, nickel, iron, cobalt and their oxides may be used as magnetic nanoparticles for drug delivery, three-dimensional cell organization, cell tracking, imaging procedures and as biosensors [16,49]. Some magnetic nanoparticles offer super paramagnetic properties with long-lasting and non-invasive tracking possibility. However, magnetic nanoparticles are cytotoxic and coating is required due to the safety concerns for overcoming their cytotoxicity and for producing biocompatible magnetic NPs [16].

#### 1.6.2. Organic Nanoparticles

##### Liposomes

Liposomes are vesicles composed of bilayers of phospholipids [50]. Their properties are affected by their lipid size, surface charge, composition and preparation method [16]. Liposomes are biodegradable, stimuli responsive and can be incorporated with hydrophilic/hydrophobic drugs [16,51]. They are mainly used in drug delivery and imaging [52]. They are the most clinically tested nanosystems, with several commercialized formulations [51]. However, their rapid clearance from circulation and scale-up issues might represent some disadvantages [16,51].

##### Natural Polymeric NPs

Natural polymers are biocompatible and biodegradable materials with bulk physical properties. They are divided mainly as proteins and polysaccharides [53] and can be listed as collagen, alginate, fibrin, gelatin and chitosan [17]. Due to their intrinsic biocompatibility and biodegradability, natural polymers are very important for bone tissue engineering procedures [54]. The synthesis methods can be flexible and polymer chains can show a wide diversity of composition and properties [53]. Generally they have high drug loading capability, but the scale-up issues might represent some disadvantages [16,53].

• Collagen

Collagen is the most frequently tested natural polymer since the ECM of the natural bone is mainly composed of collagen. It has excellent biocompatibility, optimal biodegradability and negligible immunogenicity [17,55]. The limitations of the collagen can be listed as weak mechanical stability, possible denaturation on processing and the intrinsic risk of causing immune reactions and/or transmitting infectious agents. As an alternative solution, instead of collagen extracted from animals, recombinant collagen was proposed for tissue engineering procedures [17].

• Gelatin

Gelatin is a denatured form of collagen with similar beneficial biological properties and limitations. Gelatin degradation is controllable by tailoring the crosslinking procedures. The existing presence of functional groups additionally allow functionalization by chemical transformation [17].

• Chitosan

Chitosan has many advantageous biological and physicochemical properties. It is biocompatibile, antibacterial and its production is easy. Various studies have tested chitosan as a factor and/or drug delivery system for pharmaceutical and tissue engineering applications [5,17].

Chitosan nanomaterial was tested by researchers with different combinations of nanoparticles such as PLGA/bioactive glass [1], PCL/Sr-doped calcium phosphate [56], PLGA/silver [22] and gold [48] for alveolar bone regeneration procedures with successful results in favor of alveolar bone regeneration.

• Alginate

Alginates are biocompatible, hydrophilic, non-immunogenic and cost effective materials. Their intrinsic features can induce in vivo calcification without the use of any additives and they are suitable for a number of tissue engineering and drug delivery applications [17]. However their rather slow degradation when implanted in bone defects is poorly controllable and they do not have cell attachment sites for osteoblast anchorage [17].

##### Carbon

• Carbon Nanotubes

Carbon nanotubes (CNTs) are carbon allotropes with a long cylindrical structure. They might be single-walled or multi-walled graphitic hollow tubular structures. They have mechanical and chemical stability with optimum electrical properties. However, their good chemical stability might represent a drawback for the covalent functionalization process. Carbon materials are added to the composite scaffolds, in order to reinforce the structural properties [16].

Carbon nanotubes are effective on bone tissue regeneration as they promote cell proliferation and osteogenic differentiation [57]. Several carbon nanomaterials like CNTs, graphene oxide (GO), fullerenes, carbon dots (CDs), nano-diamonds and their derivatives were successfully tested as a scaffold for bone tissue engineering [57].

• Graphene and Its Derivates

Graphene is a film monolayer one-atom-thick, with a honeycomb-like structure made of carbon atoms, and arranged in a two-dimensional hexagonal structure [58]. Nanomaterials of the graphene family include a great variety of graphene derivatives such as, graphene oxide (GO), reduced graphene oxide (rGO) and graphene nanosheets [59,60]. Graphene and its derivatives represent advantageous mechanical, electrochemical and physical properties such as, small size, large surface area, thermal stability, electrical conductivity and mechanical strength. Additionally, graphene materials may be functionalized and combined with different biomaterials and biomolecules, such as small molecules, polymers or nanoparticles, by means of covalent or non-covalent interaction [50,51,52,53,54,55,56,57,58,59,60,61]. Graphene materials are favorable for cell differentiation, proliferation and osteogenic differentiation. Additionally they are biocompatible and represent antibacterial properties. However, the behavior of graphene is dependent on its size, surface functionalization and its coating [59,60,61]. Biomedical applications of graphene oxide can produce adverse effects mostly dependent on the dose and time. Currently, the cytotoxicity of the GO material represents a challenging situation for clinical applications. The quality of graphene plays a major role since the presence of impurities can cause undesired events [62]. Interactions between body and GO are still being investigated for in vivo applications and it is most likely that their primary clinical applications shall be the ones for topical use or short-time transient implantations [63].

GOs are able to enter into cytoplasm and nuclei. They can induce cell membrane damage and apoptosis. GO can induce lung diseases by causing severe toxicity. GOs also stay for a long time in kidneys since they are cleaned in the kidney by a very complex process [64]. Further studies are lacking in the literature to explore their toxicity and effect on cells/tissues.

Graphene oxide combined with silk fibroin [65,66], GO-coating of titanium implants [67] and GO-coating of collagen membranes [60,61] for guided bone regeneration were investigated by various researchers. The results showed that these applications can improve cell proliferation and osteogenic differentiation with success.

#### 1.6.3. Composite Scaffolds

Inspired by the natural organic/inorganic composition of bone, different organic and/or inorganic particle combinations were tested as composite scaffolds that increase the advantages and decrease the drawbacks of each component [17]. In order to overcome the disadvantages of a single particle, strategies of different composite scaffold designs containing diverse materials have been developed by many researchers [68].

Combining nHA with polymers of high molecular weight was evaluated to overcome the disadvantages of a single material. PLA [19], PLGA [69], collagen [70], polyamide [71], coralline [72], chitosan and PCL [73] have been combined with nHA and were found to improve scaffold biocompatibility as well as mechanical strength. As an example for bone regeneration, several studies have shown that the combination of nHA and collagen scaffolds has favorable mechanical properties [19,70]. Collagen fiber provides an osteoinductive and absorbable scaffold for infiltration of osteoblast cells. However, the nonabsorbable nHA can reduce this effect. For this reason, the percentage of nHA and collagen in composite materials play a crucial role for tissue regeneration [19].

### 1.7. Manufacturing Methods to Fabricate Scaffolds

Strategies of scaffold designs utilizing particles can be listed as follows: (i) Particles as different single components embedded into matrices, solid polymers, hydrogels or calcium phosphate cements, (ii) particles combined into blocks for fabrication of scaffolds, (iii) haphazard packing, (iv) rapid prototyping and self-assembling scaffolds (assembly driven by electrostatic, magnetic interactions or hydrophobic interactions) [17].

Various methods have been developed to produce scaffolds with porous properties for bone regeneration, such as freeze-drying [74], gas foaming [75], salt leaching [76], phase transformation [77], sponge replication [78], electrospinning [79] and additive manufacturing methods such as 3D printing (selective laser sintering (SLS), stereolithography, laser assisted printing, inkjet printing and extrusion printing) [32,68,80].

The sol-gel phase, co-precipitation as well as chemo-mechanical techniques have been utilized for nHA production [81]. However, these procedures are complex with high cost and poor reproducibility. Additionally, the manufacturing variables, the low-yield end-products and high byproducts are difficult to control, and the produced nHA is different from the biological HA in terms of physicochemical properties [81].

### 1.8. Biphasic and Multiphasic Scaffolds

The nanoparticles have many limitations such as a high agglomeration rate and an uneven distribution pattern. Biphasic and multiphasic preparations might partially overcome the limitations of single-phase biomaterials by improving the bioactivity and biocompatibility [32] since they have the ability to combine different material compositions and physical structures [82,83,84].

The rationale of utilizing multiphasic/biphasic scaffolds in oral science mostly depends on the expectations from an ideal scaffold. An ideal scaffold should have various functions, such as the support for cell colonization, migration, differentiation and growth. The design should also take into account suitable physical and physicochemical properties together with degradation kinetics. Multiphasic scaffolds are needed for an appropriate control of the spatiotemporal events for periodontal tissue regeneration since they can allow compartmentalized tissue healing [82,83]. Manufacturing of multiphasic nanoscaffolds for periodontal tissue engineering can be divided into three approaches: (i) Bilayered occlusive membrane + bone compartment [84,85], (ii) compartmentalized biphasic scaffolds [86,87,88,89] and (iii) compartmentalized triphasic scaffold [90]. In vitro and in vivo investigations have reported promising results for biphasic/multiphasic scaffold designs. However, the methods still need to be refined for future clinical applications.

### 1.9. Three-Dimensional (3D) Porous Scaffolds

Three-dimensional porous scaffolds have been developed and tested in many tissues, including bone, cartilage, skin, muscle and vasculature [17]. The importance of 3D porous scaffolds comes from their ability to simulate extracellular matrices, which can sustain cellular activity. They can provide a pool of bioactive signaling molecules through a sustained release to the natural environment. In this way, they might regulate cell function and trigger tissue repair [17].

### 1.10. Three-Dimensional Bio-Printing

Three-dimensional bio-printing (3D additive manufacturing) is a precise layering of biomaterials, biochemical substances and living cells, with spatial control of the positioning of each component [91]. Tissue bio-printing strategies include inkjet, micro-extrusion and laser-assisted bio-printing. Three-dimensional printing is one of the hot topics in TE/RM and nanomaterials play a crucial role in this manufacturing method [92,93]. The major challenge in these technologies is to provide multiple cell types and micro-architecture of extracellular matrix (ECM) in an adequate resolution to reproduce biological function [92,93].

In periodontal regeneration, the main challenge is the scaffold design, which might mimic the periodontal tissue nature and organization. While 3D bio-printing may help to solve this challenge, the long-term success of these applications mostly depends on the properties of the current biomaterials [82,94].

### 1.11. Influence of Magnetic Fields on Biological Systems

Magnetism might play a significant role in the control of cell responses. A magnetic field can be applied to cells externally or internally; cells can be embedded in a scaffold material with magnetic properties [95]. Yun et al. investigated the additional effects of the outer static magnetic field (SMF) with magnetic polycaprolactone nanocomposite scaffold for bone regeneration. According to the results of this experiment, the combination of external and internal magnetism can be advantageous for new bone formation [95].

## 2. Nanoscaffold Applications for alveolar Bone Regeneration

In TE/RM, one of the major research topics is to create bioconstructs that can integrate with the in vivo tissues [10]. Current systems represent limitations such as restricted diffusion and uneven cell-matrix distribution. In order to overcome limitations of the current materials, during the last years different types of scaffolds and bioreactors have been designed and tested [10]. The regeneration of oral tissues is very challenging [96] and nanomaterials might represent a great potential for future TE/RM applications of various craniofacial and oral defects. The most frequently investigated nanomaterials for oral tissues can be listed as nanofibers, nanoparticles, nanosheets, nanotubesand nanospheres. Recently, the multi-layer nanoscaffolds for oral tissue regeneration were also tested by researchers [97,98].

In general, nanomaterials have excellent physiochemical properties and biomimetic features for enhancing cell growth and tissue regeneration [96,97]. Nanomaterials, due to their size, can have an effective control of the release rate of the each agent upon matrix degradation. Furthermore, they can be used for delivery of therapeutic agents in alveolar bone and tooth regeneration. Nanofibers represent an ideal environment for cell migration due to their similarity to extracellular matrices and their high porosity. In addition, nanotubes and nanoparticles might enhance the chemical and mechanical features of the scaffold, increase cell migration and proliferation, and tissue regeneration [96]. While the application of nanomaterials in TE/RM is still in its infancy phase with several limitations to be addressed, the recent results of investigations indicate their potential role for future oral tissue engineering applications [93].

Three fabricating modes exist for TE/RM scaffolds: Growth factor-scaffold, cell-scaffold and cell growth factor-scaffold. In cell-scaffold, and cell growth factor-scaffold materials, cells are inserted on a biocompatible scaffold, in order to proliferate into the scaffold and to remodel the natural environment by secreting ECM. Biomaterials in periodontal tissue engineering are mainly growth factor-scaffold, used as the carrier of exogenous growth factors. The main challenge is the necessity to provide an adequate number of seeded cells integrated with the scaffold material in vitro and implanted into a defect position [96,99].

There is a huge number of experimental studies reported in the literature, searching for the best nanoscaffold material for various periodontal tissue regeneration applications. Ogawa et al. [41] tested nano-ß-TCP/collagen loaded with fibroblast growth factor-2 (FGF-2) on periodontal wound healing. In the study, nano-ß-TCP scaffold, nano-ß-TCP scaffold loaded with FGF-2 and non-coated collagen scaffold were implanted into one-wall infrabony defect models. nano-ß-TCP scaffold was fabricated by surface coating of collagen scaffold with nanosize ß-TCP dispersion. According to the four week post-surgery histological results, nano-ß-TCP scaffold loaded with FGF-2 displayed nearly five-times greater periodontal tissue repair when compared with the collagen scaffold [41].

Xue et al. [22] produced nanoparticles of chitosan, PLGA and silver (Ag) for investigating the optimal combination ratio for mineralization of periodontal membrane cells, and periodontal tissue regeneration. The antibacterial properties of each nanoparticle were also evaluated. Different combination ratios of nPLGA and chitosan were tested. According to the results, single nanoparticles did not show any cytotoxicity and were able to enhance cell mineralization. Additionally, chitosan and nAg showed antibacterial properties, while nAg limited cell proliferation. The 3:7 ratio of nPLGA/chitosan and 50 μg/mL nAg was the optimal proportion [22].

Hydrogels are soft materials with (3D) polymer structure, water-absorbability and adjustable physical and chemical properties [100]. Natural and synthetic hydrogels composed of micro-/nanostructures have been shown to imitate the chemical and physical properties of natural ECM for bone and periodontal tissue regeneration [37,38,39,40,41,42,43,44,45,46,47,48,49,50,51,52,53,54,55,56,57,58,59,60,61,62,63,64,65,66,67,68,69,70,71,72,73,74,75,76,77,78,79,80,81,82,83,84,85,86,87,88,89,90,91,92,93,94,95,96,97,98,99,100,101]. Gelatin can be modified by methacryloyl (methacrylamide and methacrylate) side groups and the final product is GelMA [84]. Gelatin methacrylate (GelMA) is a biocompatible and photocrosslinkable hydrogel. Chen et al. [37] tested fabrication of GelMA/nanohydroxylapatite microgel arrays using a photocrosslinkable method. The group evaluated the regeneration and osteogenic proliferation of human periodontal ligament stem cells (hPDLSCs) encapsulated in microgels. According to the results GelMA/nHA microgels (10%/2% w/v) enhanced periodontal tissue regeneration. Since GelMA/nHA microgels enhanced hPDLSCs proliferation and osteogenic differentiation in vitro and supported bone regeneration in vivo [37].

In recent years, researchers proposed and tested graphene-based nanomaterials for oral tissue engineering because of the many advantageous properties and antibacterial capacities of the graphene [60,61,102].

Sowmya et al. [1] tested a scaffold system for the regeneration of oral tissues (periodontal ligament, cementum and alveolar bone) in a rabbit study [1]. The structure of the scaffold consisted of three layers. For bone tissue a layer of chitosan/PLGA/nano-sized bioactive glass layer enriched with PRP, for periodontal ligament a layer of chitosan/PLGA with the adjunct of fibroblast growth factor 2 (FGF-2), and finally for cementum a layer of chitosan/PLGA/nano-sized bioactive glass layer loaded with cementum protein 1 (CEMP1). According to the histological and tomographic evaluation results, new alveolar bone formation and complete periodontal healing was obtained in three months [1].

Zhang et al. [103] tested the incorporation of growth factors in nanomaterial-based silk fibroin scaffolds in a dog study for tissue regeneration. Nanomaterial-based silk fibroin scaffolds loaded with BMP-7 and/or platelet derived growth factor (PDGF)-ß adenovirus were used to test tissue regeneration. According to the results, the scaffolds loaded with BMP-7 mainly enhanced alveolar bone regeneration, while the scaffolds incorporated with PDGF-ß adenovirus produced a partial regeneration of the periodontal ligament. The combination of growth factors created a synergistic effect showing up to two times greater alveolar bone, periodontal ligament, and cementum formation, as compared with each factor alone [103].

Vaquette et al. [40] tested PCL and ß–TCP for periodontal ligament and alveolar bone regeneration. The scaffold design was composed of a flexible electrospun component for the periodontal ligament section and a fused deposition modeled component for the bone section. Cell sheet technology was utilized to manufacture biphasic scaffolds additively. While the results were promising with increased mechanical stability of the cell sheets and mineralization, the bone section did not provide sufficient ectopic bone proliferation [40].

Rasperini et al. [104] tested an individualized custom-made 3D-biomanufactured scaffold for regeneration of a periodontal osseous defect in a clinical study. For this purpose, a customized scaffold containing PCL powder and HA was produced by using selective laser sintering technology, according to the computed tomography scan of the patient’s defect. While the scaffold became exposed after 12 months, which led to failure, the results of this study are promising in terms of further research [104].

Nanomaterials seem to be promising for alveolar bone regeneration applications as they mostly provide favorable results, as listed in Table 1. However, the decreased size of nanomaterials can cause some potential problems. Nanomaterials possess sizes close to biological molecules, peptides, deoxyribonucleic acid and viruses. Thus, they can provoke adverse events by moving throughout the body, depositing in target organs (liver, lungs, kidney, heart and spleen), penetrating cell membranes and staying in the mitochondria [105,106,107,108]. Manganese oxide, titanium dioxide, aluminum oxide, zinc oxide and silver might accumulate and elicit harmful responses [109]. The brain is partly shielded by the blood–brain barrier, but the nanoscale size of these materials might cause them to cross this barrier or they might penetrate through the olfactory and sensory nerves. The biological toxicity of nanomaterials is thought to be induced by their oxidative properties [110,111] and further research should be conducted to evaluate and develop a science-based risk evaluation of the nanomaterials [109].

One of the limitations of the nanoscaffold systems is the incorporation of nanoscale material for reinforcement. In the regeneration of bone, utilizing micro- and nano-length scale materials can be beneficial for increasing the intrinsic fracture resistance of the bone by affecting bone strength and supporting plasticity. However, macro-length scale materials are also beneficial, influencing toughness and creating a resistance to fracture. In brief, nanoscale materials increase the capacity to withstand pressure, but toughness drops dramatically [6] and it is not realistic to expect both high strength and high toughness properties from one single material [113]. As an example, in ceramic based materials, toughness is provided majorly by their large scales. Ceramic based materials are able to shield crack by supporting uncracked material bridging the crack wake, which is impossible for micro/nano-scale materials [114,115]. Nanotechnology alone cannot be the optimum solution for improving the mechanical properties of the scaffolds. Incorporation of different scale materials with a hierarchical design exhibiting many scales seems to be beneficial and should be considered as a future perspective [6,116].

## 3. Conclusions

This review mainly focused on the basic information about the nanomaterials for bone regeneration to give researchers a general knowledge about their possible applications in the oral field. Currently their application in oral and maxillofacial clinics is very limited but interest is increasing for their possible applications in order to overcome the current problems associated with conventional materials.

As a conclusion, it can be underlined that nanomaterials have excellent physico-chemical properties and biomimetic features for promoting cell growth and stimulating tissue regeneration, and oral tissue engineering with nanomaterials seems to represent a great potential with vital importance as future treatment modalities.

However, nanomaterials should not be evaluated as optimum solutions for every current problem. It should be understood that there are also disadvantages of nanomaterials such as toughness, which can be solved with the incorporation of a hierarchical design encompassing many length-scales to generate stronger and tougher scaffold materials. They can also provoke adverse events by moving all over the body or by depositing in organs due to their nanosize, which is similar to biological molecules and viruses. Further research needs to be done in order to provide solutions for possible future applications.

## Figures and Tables

**Table 1:** Nanoscaffold applications for alveolar bone regeneration.

Material	Reference	Outcome
3 layer chitosan/PLGA/nano-sized bioactive glass	Sowmya et al. [1]	Complete periodontal healing and new alveolar bone deposition after three months
GO-coating of collagen membranes	Radunovic et al. [60]	Favourable on promoting osteoblastic differentiation process
GO-coating of collagen membranes	De Marco et al. [61]	Improved biocompatibility of collagen membranes on in vitro human primary gingival fibroblast model
PCL containing ß–TCP	Vaquette et al. [40]	Enhanced mechanical stability of the cell sheets, and mineralization. However, ectopic bone ingrowth was not sufficient
nano-ß- TCP/collagen scaffolds	Ogawa et al. [41]	nano-ß-TCP/collagen scaffolds loaded with fibroblast growth factor-2 (FGF-2) improved periodontal tissue wound healing results
Chitosan, PLGA, and silver (Ag) nanoparticles complex	Xue et al. [22]	Contributed to cell mineralization without cytotoxicity
GelMA/nHAmicrogels	Chen et al. [37]	Promoted in vivo osteogenesis of hPDLSCs encapsulated in microgels
nanomaterial-based silk fibroin scaffolds incorporating BMP-7 and/or PDGF-ß	Zhang et al. [103]	Promoted periodontal healing
PCLpowder containing HA	Rasperini et al. [104]	Clinical study with failure due exposure of the scaffold
Graphene	Xie et al. [112]	Favourable on osteogenic differentiation but not on osteoblastic differentiation
Graphene Oxide combined with silk fibroin	Rodríguez-Lozano et al. [65]	Favourable on mechanical resistance and hPDLSC proliferation and showed biocompatibility
Graphene Oxide combined with silk fibroin	Vera-Sánchez et al. [66]	PDLSC proliferation rate into osteo/cementoblast like cells improved with these combinations
GO-coating of titanium implants	Ren et al. [67]	Improved cell proliferation, osteogenic differentiation and biocompatibility of implants
Citric Acid-Based Nano Hydroxyapatite	Dayashankar et al. [39]	Significant bone regeneration
Nano-bioactive glass loaded with NELL1 gene	Zhang et al. [31]	Good osteoconductivity for promoting the formation of new alveolar bone tissue
Poly(l-lactic acid) (PLLA) nanofibrous spongy microspheres, PLLA/polyethylene glycol (PEG) co-functionalized mesoporous silica nanoparticles, and poly(lactic acid-*co*-glycolic acid) (PLGA) microspheres	Liu et al. [23]	In a mouse model of periodontitis, the injectable and biomolecule-delivering PLLA lead to Treg enrichment, expansion, and Treg-mediated immune therapy against bone loss
Nanofibrous yarn reinforced HA-gelatin	Manju et al. [34]	Promoted bone formation in critical sized alveolar defects in rabbit model
Silver nanoparticle-coated collagen membrane	Chen et al. [35]	Induced osteogenic differentiation of mesenchymal stem cells that guide bone regeneration.
Chitosan-gold nanoparticles mediated gene delivery	Takanche et al. [48]	Enchanced osseointegration of dental implant even in osteoporotic condition
Hydroxyapatite nanowires modified polylactic acid membrane	Han et al. [36]	Promoted bone regeneration in a rat mandible defect model
PCL/chitosan/Sr-doped calcium phosphate electrospun nanocomposite	Ye et al. [56]	Higher ALP activity level and a better matrix mineralization
Nano hydroxyapatite mineralized silk fibroin	Nie et al. [38]	Improved osteogenesis
PLGA/PCL Modification Including Silver Impregnation, Collagen Coating, and Electrospinning	Qian et al. [24]	Enhanced alveolar bone regeneration (31.8%)

## Abbreviations

TE/RM	Tissue engineering and regenerative medicine
GF	Growth factors
BMP	Bone morphogenetic proteins
mRNA	Micro ribonucleic acid
TCP	Tricalcium phosphate
BMMSC	Bone marrow mesenchymal stem cells
MSC	Mesenchymal stem cells
PRP	Platelet rich plasma
ECM	Extracellular matrix
nm	Nanometers
µm	Micrometers
NP	Nanoparticles
PLA	Poly-lactic acid
PGA	Poly-glycolic acid,
PLGA	Poly-lactic-co-glycolic acid
PLLA	poly(l-lactic acid)
PEG	PLLA/polyethylene glycol
PCL	Poly-caprolactone
CP	Calcium Phosphate
HA	Hydroxyapatite
TCP	Tricalcium phosphate
nHA	Nano hydroxyapatite
nTCP	Nano tricalcium phosphate
MTA	Mineral trioxide aggregate
MSNP	Mesoporous silica nanoparticle
CNT	Carbon nanotube
GO	Graphene oxide
CD	Carbon dots
GO	Graphene oxide
rGO	Reduced graphene oxide
SLS	Selective laser sintering
3D	3 dimensional
SMF	Static magnetic field
FGF-2	Fibroblast growth factor-2
Ag	Silver
GelMA	Gelatin methacrylate
hPDLSC	Human periodontal ligament stem cells
CEMP1	Cementum protein 1
PDGF	Platelet derived growth factor
DNA	Deoxyribonucleic acid
